# In the preparation and administration of intravenous medicines, what are the best practice standards that healthcare professionals need to follow to ensure patient safety? Protocol for a systematic review.

**DOI:** 10.12688/hrbopenres.13028.2

**Published:** 2020-12-10

**Authors:** Peter J. Carr, Laura O'Connor, Georgina Gethin, John D. Ivory, Paul O'Hara, Orla O'Toole, Patricia Healy

**Affiliations:** 1 School of Nursing and Midwifery, National University of Ireland Galway, Galway, Ireland; 2 University Hospital Galway, Galway, Ireland; 3 Clinical Trials Unit, HRB Clinical Research Facility, University Hospital Galway, Galway, Ireland

**Keywords:** intravenous therapy and medicine, review protocol, evidence synthesis, patient safety, practice standards

## Abstract

**Introduction:** Intravenous therapy and medicines (IVTM) are the most common invasive interventions in use in healthcare. Prescribed IVTM play an essential role in the treatment of illness, management of chronic conditions and in maintaining health and wellbeing. The intravenous (IV) route is the administration of concentrated medications (diluted or undiluted) directly into peripherally or centrally inserted vascular access devices. Medication safety is a key priority and best practice standards are required to guide the safe preparation and administration of IVTM.

**Methods:** We will conduct a systematic review of the literature pertaining to the preparation and administration of intravenous therapy and medicines. Our search will include studies concerned with the preparation and/or administration of IVTM via peripheral or central vascular access devices. We will be guided by the preferred reporting items for systematic review and meta-analysis (PRISMA) in this review. Literature will include all trial designs, national/international guidelines, and expert consensus opinion made available in English from 2009 to present day.

**Conclusions:** We will synthesise the evidence concerning safe and effective preparation and administration of intravenous therapy and medicines to inform the development of a national guideline for healthcare professionals in Ireland. The availability of up-to-date, contemporaneous evidence-based practice standards will ensure quality and safety for service-users.

**Registration:**  This study has been submitted to PROSPERO and we are awaiting confirmation of registration.

## Introduction

While the concept and practice of intravenous therapy is centuries old, with the first documented blood transfusion taking place in 1492, the key advancements that have made it a ubiquitous part of healthcare today largely occurred in the last two centuries ^1^. The cholera epidemic in the early 19 ^th^ century led Dr William Brooke O'Shaughnessy to state “ *I would not hesitate to inject some ounces of warm water in the veins. I would also, without apprehension, dissolve in that water the mild innocuous salts which nature herself is accustomed to combine with the human blood*” ^2^, a theory later tested by Dr Thomas Latta to great success ^3^. Since then healthcare has witnessed a proliferation of new medicines requiring administration via the circulatory system.

Medication safety has been identified internationally by the World Health Organisation as a key area for improvement in all healthcare settings ^4^. In Ireland, a number of medication-related errors appear in the top ten medical incidents reported to the State Claims Agency ^5^ (for example, incorrect dosages, missed medications, medications being incorrect/not reconciled for changes in care). Such medication incidents were reported from multiple services nationally ^6^. Intravenous therapy and medicines (IVTM) safety can impact greatly on patient care. High profile medication errors have directly led to guidance being developed, as well as landmark legal rulings, both resulting in widespread changes to practice ^7^.

With up to 90% of admitted patients now receiving IVTM at some point during their stay in hospital, relevant guidelines are more necessary than ever ^8^. Additionally, while administration of IVTM was initially considered a role for the doctor alone, rapid developments in the field and the changing roles of healthcare professionals (HCPs) means that the majority of clinical staff will be responsible for the management, preparation, and administration of IVTM at some point. This highlights the various number of healthcare professionals involved in IVTM.

In 2018, the Irish Health Information and Quality Authority (HIQA) published its recommendations on the first phase of the Medication Safety Monitoring Programme. It recommends that hospitals provide clinical staff with easily accessible information such as policies, procedures, guidelines and/or protocols to guide the safe use of medicines at the point of prescribing, preparation and administration ^9^.

A guideline is defined as “a principle of criterion that guides or directs action” ^10^. The Irish Health Service Executive framework for developing guidelines emphasises using clear evidence from the existing literature, rather than expert opinion alone ^10^. In systematically reviewing available literature, we can provide better knowledge and evidence for robust guideline for HCPs.

The aim of this review is to synthesise the evidence for a specific question: *In the preparation and administration of intravenous medicines, what are the best practice standards that healthcare professionals need to follow to ensure patient safety?*

This evidence synthesis will then feed into the development of new guidelines for the Irish Health Service Executive (HSE) and be disseminated to the wider research and clinical community. Our report will be guided by the PRISMA statement checklist (preferred reporting items for systematic review and meta-analysis) and the synthesis without meta-analysis (SWiM) extension ^11, 12^.

### Objective

Our primary research question, devised by the HSE to inform their guideline development, is “In the preparation and administration of intravenous medicines, what are the best practice standards that healthcare professionals need to follow to ensure patient safety?”. The working group developing this national guideline have further identified the following areas as specific topics of interest:

- Literature relating to independent double checking of IVTM
- Literature relating to the practices required to ensure complete administration of IVTM
- Literature relating to use of infusion pumps for the delivery of IVTM
- Literature relating to the standards required for labelling IVTM
- Literature relating to the education preparation and competency requirements for healthcare professionals administering IVTM
- Literature relating to the involvement of the following undergraduate students in the process of preparation and administration of IVTM: nurses, midwives, doctors, paramedics and radiographers.

## Methods

### Criteria for inclusion of study designs

To ensure that all relevant national and international peer reviewed evidence and policy literature is considered, this review will include randomised controlled trials (RCTs), systematic reviews, meta-analyses, cohort studies, observational studies, national/international guidelines, and expert consensus opinion made available from 2009 to current (See Table 1 for inclusion and exclusion criteria).

**Table 1.** Inclusion and exclusion criteria.

Included	Excluded
RCTs, systematic reviews, meta-analyses, cohort studies, observational studies, national/international guidelines, and expert consensus opinion	Studies published before 2009
Studies involving the preparation and/or administration of IV medicines via peripheral or central venous access devices	Studies focusing on needlefree devices or the introduction, care, or maintenance of access devices
Studies focusing on medical professionals, nurses, midwives, paramedics and radiographers, working within pre-hospital, acute hospital and community settings	Studies examining the administration of blood, blood products, or parenteral nutrition
Studies carried out in pre-hospital, acute hospital and community settings	Studies focusing on consent to IVT treatment or infection prevention

RCTs, randomised controlled trials.

### Inclusion

Literature must involve the preparation and/or administration of IV medicines via peripheral or central venous access devices.

### Exclusion

Studies focusing on needlefree devices or the introduction, care, or maintenance of access devices will be excluded, as will studies examining the administration of blood, blood products, or parenteral nutrition. Studies focusing on consent or infection prevention will be also be excluded, owing to the robust guidelines already integrated into practice (e.g. Epic3 guidelines ^13^ and the Aseptic Non-Touch Technique (ANTT) framework ^14^). Work published before 2009 will be excluded, to ensure the synthesis reflects current best practices.

### Participants

All patients receiving IV medicines (adults, children and neonates) in primary or secondary care settings (i.e. pre-hospital, acute hospital and community settings).

### Interventions

We will consider for inclusion any publication that details the preparation of IVTM for administration via peripheral or central venous access devices. This will include slow bolus IVTM injections, intermittent and continuous IV infusions. This review will include studies focusing on medical professionals, nurses, midwives, paramedics and radiographers, working within pre-hospital, acute hospital and community settings.

### Outcomes

This review will gather safety and effectiveness outcome data. As these two terms underpin best clinical practice, we propose a broad definition of the following:

1. For safety outcomes, we will include papers with outcomes relating to incidences of errors in the preparation and/or administration of IVTM.
  a. *We will extract data relating to the effectiveness of the intervention in question, i.e. the outcomes as chosen within each study*
2. For effectiveness outcomes:
  a. *We will extract data relating to the effectiveness of the intervention in question, i.e. the primary outcomes as chosen within each study*
  b. We will extract data from studies which describe effectiveness of practice

### Search strategy

We will search the following databases for material published after 2009: Cochrane Library, PubMed, CINAHL, and Web of Science. Additionally, we will search OpenGrey and OAlster for grey literature. An example of the search devised for CINAHL is available as *Extended data* ^15^. Searches will be designed by an appropriately experienced search methodologist team member (JDI).

### Study screening

Titles and abstracts will be imported into the systematic review software Covidence ^16^. Two authors will screen the title and abstracts of the citations against the pre-specified eligibility criteria. Any discrepancies will be resolved by a content-expert author (PC) if consensus cannot be reached. The same process will be followed for full-text screening. We will record a rationale for exclusion for any papers deemed ineligible at full text. Both title and abstract and full-text screening processes will be piloted to ensure consistency across authors.

### Data extraction

Data will be extracted from included studies by two reviewers (PC, LOC) independently using the data extraction tool within Covidence. Any discrepancies between reviewer’s extractions will be highlighted by Covidence, and consensus will be reached through discussion where necessary. The data extraction form will be piloted by two authors (PC, LOC).

Data extracted from each study (where provided) will include:

- Primary outcome – All reported outcomes, primary and secondary relating to our defined outcomes of safety and effectiveness
- Study description: design, methodology (e.g RCT, cohort study), methods of analysis, data type (qualitative/quantitative), publication date, clinical trial registration and study protocol.
- Health care personnel and study demographics: e.g role and treatment setting
- Care characteristics: primary disease, treatment(s), duration of care
- IVTM treatment details: drug administered, frequency, duration, access device used

As our primary aim is to narratively synthesis the evidence, we will not be extracting data relating to risk of bias at this time. However, during data extraction, reviewers will take notes relating to methodology and study quality, to provide a rudimentary indication of evidence quality, and to inform potential further analyses.

### Data synthesis

There are two phases to our planned data synthesis. Firstly, we will narratively synthesise the included full text papers with respect to the specific objectives and report these findings.

Secondly, should sufficient suitable studies be found, additional separate quantitative and qualitative analyses will be carried out.

### Summary of finding and data visualisation

We will produce a summary of findings addressing the focused objectives listed above. We anticipate high levels of heterogeneity in eligible studies thus ruling out the possibility of meta-analysis. Therefore, the Synthesis Without Meta-analysis (SWiM) extension to the PRISMA statement checklist will guide the optimal reporting of the work and results ^12^.

### Dissemination

A report summarising all major findings aligned with the focused objectives will be provided to the HSE to inform their guideline development. Some dissemination activities will be focused specifically on this audience (e.g. summary infographics, invited talks, etc.). Additionally, we plan to publish our findings in a peer reviewed journal. Should the data allow, we will separate qualitative and quantitative evidence and publish syntheses of both.

## Current study status

The search for this study was carried out in February 2020. Title and abstract screening was completed in early March, and full text screening is expected to be completed by early April.

## Conclusion

This review will synthesise best available evidence for the preparation and administration of IVT as demonstrated in existing guidelines, peer-reviewed science, and expert consensus, forming an overview of the current state of the field. Ultimately, the findings will be used to inform the development of a Health Service Executive (HSE) national guideline for the administration of intravenous medications by healthcare professionals in Ireland. As such, a limitation of the study is the specificity of the inclusion and exclusion criteria, which are designed to allow this work to complement existing syntheses and minimise overlap with guidelines that are already integrated into clinical practice.

## Data availability

### Underlying data

No underlying data are associated with this article.

### Extended data

Open Science Framework: In the preparation and administration of intravenous medicines, what are the best practice standards that healthcare professionals need to follow to ensure patient safety? https://doi.org/10.17605/OSF.IO/U3JBW ^15^.

### Reporting guidelines

Open Science Framework: PRISMA-P checklist ^17^ for “Study Protocol: In the preparation and administration of intravenous medicines, what are the best practice standards that healthcare professionals need to follow to ensure patient safety?”. https://doi.org/10.17605/OSF.IO/U3JBW ^15^.

Data are available under the terms of the Creative Commons Attribution 4.0 International license (CC-BY 4.0).
