# Supplementary material for: In the preparation and administration of intravenous medicines, what are the best practice standards that healthcare professionals need to follow to ensure patient safety? Protocol for a systematic review
Source: HRB Open Res. 2021 May 12;3:19. Originally published 2020 May 1. [Version 3] doi: 10.12688/hrbopenres.13028.3 (PMC8167500; doi:10.12688/hrbopenres.13028.3)
Supplement: Peer review history

# Peer review: Reviewer response for version 3

**DOI:** 10.21956/hrbopenres.14473.r29414

**Published:** 2021-05-28

**Authors:** Zane Robinson Wolf

**Affiliations:** 1 Nursing Program, La Salle University School of Nursing and Health Sciences, Philadelphia, Pennsylvania, USA

I appreciate the changes and approve the plan/paper.

Is the study design appropriate for the research question?

Yes

Is the rationale for, and objectives of, the study clearly described?

Yes

Are sufficient details of the methods provided to allow replication by others?

Partly

Are the datasets clearly presented in a useable and accessible format?

Not applicable

Reviewer Expertise:

Intravenous medication errors best practices

I confirm that I have read this submission and believe that I have an appropriate level of expertise to confirm that it is of an acceptable scientific standard.

# Peer review: Reviewer response for version 3

**DOI:** 10.21956/hrbopenres.14473.r29415

**Published:** 2021-05-12

**Authors:** Ariane Ferreira Machado Avelar

**Affiliations:** 1 Department of Pediatric Nursing, Escola Paulista de Enfermagem, Universidade Federal de São Paulo, São Paulo, Brazil

Manuscript approved. Congratulations!

Is the study design appropriate for the research question?

Yes

Is the rationale for, and objectives of, the study clearly described?

Yes

Are sufficient details of the methods provided to allow replication by others?

Partly

Are the datasets clearly presented in a useable and accessible format?

Not applicable

Reviewer Expertise:

Infusion therapy, peripheral intravenous access, sleep, ambience, neonatal nursing, pediatric nursing.

I confirm that I have read this submission and believe that I have an appropriate level of expertise to confirm that it is of an acceptable scientific standard.

# Peer review: Reviewer response for version 2

**DOI:** 10.21956/hrbopenres.14251.r28535

**Published:** 2021-02-10

**Authors:** Ariane Ferreira Machado Avelar

**Affiliations:** 1 Department of Pediatric Nursing, Escola Paulista de Enfermagem, Universidade Federal de São Paulo, São Paulo, Brazil

I appreciate the opportunity to review the manuscript and consider the revisions appropriated and approved.

Is the study design appropriate for the research question?

Yes

Is the rationale for, and objectives of, the study clearly described?

Yes

Are sufficient details of the methods provided to allow replication by others?

Partly

Are the datasets clearly presented in a useable and accessible format?

Not applicable

Reviewer Expertise:

Infusion therapy, peripheral intravenous access, sleep, ambience, neonatal nursing, pediatric nursing.

I confirm that I have read this submission and believe that I have an appropriate level of expertise to confirm that it is of an acceptable scientific standard.

# Peer review: Reviewer response for version 2

**DOI:** 10.21956/hrbopenres.14251.r28705

**Published:** 2021-01-22

**Authors:** Zane Robinson Wolf

**Affiliations:** 1 Nursing Program, La Salle University School of Nursing and Health Sciences, Philadelphia, Pennsylvania, USA

The research questions and study methods are appropriate.

 Specify the search terms and that terms might be added during the search process.

 Although opinion articles and grey literature will be appraised, unpack this.

 Identify column headings for the appraisal matrix.

 Providers have had experiences whereby health care institutions changed vendors leading to errors. Purchasing is pre-prescription.

Is the study design appropriate for the research question?

Yes

Is the rationale for, and objectives of, the study clearly described?

Yes

Are sufficient details of the methods provided to allow replication by others?

Partly

Are the datasets clearly presented in a useable and accessible format?

Not applicable

Reviewer Expertise:

Intravenous medication errors best practices

I confirm that I have read this submission and believe that I have an appropriate level of expertise to confirm that it is of an acceptable scientific standard, however I have significant reservations, as outlined above.

# Response

**Published:** 2021-04-16

**Authors:** Laura O'Connor

**Affiliations:** National University of Ireland, Galway, Ireland

Thank you for the time you have taken to read and review our protocol. Below we reply to your comments to make clear the changes we have made in light of your review.

*Specify the search terms and that terms might be added during the search process.*

 Our search strategy was extensive, and as such a sample of the strategy for one database was included as extended data. We have now updated this extended data folder to add other search strategies where it was possible to export them from the database or recreate them.

*Although opinion articles and grey literature will be appraised, unpack this.*

 Further detail on appraisal of disparate types of literature added

*Identify column headings for the appraisal matrix.*

 We have expanded the Data Synthesis section to include a clearer explanation of the headings to be used when appraising included papers.

*Providers have had experiences whereby health care institutions changed vendors leading to errors. Purchasing is pre-prescription.*

 We agree that the preparation and administration of intravenous medicines includes other processes that we do not cover. The scope of this review has been defined by a specific need of a department of the Irish Health Service Executive to answer a narrow question. As such, this review’s focus is primarily on those preparation and administration steps which happen close to or at the bedside.

# Peer review: Reviewer response for version 1

**DOI:** 10.21956/hrbopenres.14123.r27569

**Published:** 2020-07-09

**Authors:** Ariane Ferreira Machado Avelar

**Affiliations:** 1 Department of Pediatric Nursing, Escola Paulista de Enfermagem, Universidade Federal de São Paulo, São Paulo, Brazil

Medication safety is one of the greatest indicators of health care quality. Errors related to intravenous therapy process are among the most frequent worldwide, and research into which group the best practice standards during the preparation and administration of intravenous medicines are essential to promote patient safety.

 The study protocol is well designed and clearly justified. I have a few comments which I hope will refine the manuscript.

 The title of the manuscript must contain information that deals with a systematic review.

 The research question is clear and well delimited, and the main objective is to synthesize the evidence of the best practices that health professionals should follow during the preparation and administration of intravenous medicines, to feed the development of new guidelines for the Irish Health Service Executive.

 The topic of interest showed in the Objectives topic: “Literature relating to the involvement of the following undergraduate students in the process of preparation and administration of IVTM: nurses, midwives, doctors, paramedics and radiographers” does not seems to be suitable to answer the research question. I suggest delimiting the search only in the best practices of preparation and administration of medicines, regardless of the professional who performs it.

 The inclusion criteria “Studies focusing on medical professionals, nurses, midwives, paramedics and radiographers, working within pre-hospital, acute hospital and community settings” does not seems to be suitable. The objective of this study is the best practices for the preparation and administration of drugs and solutions, which must be followed by all professionals, regardless of the professional category.

 The search strategies must be clarified. What descriptors will be used?

Is the study design appropriate for the research question?

Yes

Is the rationale for, and objectives of, the study clearly described?

Yes

Are sufficient details of the methods provided to allow replication by others?

Partly

Are the datasets clearly presented in a useable and accessible format?

Not applicable

Reviewer Expertise:

Infusion therapy, peripheral intravenous access, sleep, ambience, neonatal nursing, pediatric nursing.

I confirm that I have read this submission and believe that I have an appropriate level of expertise to confirm that it is of an acceptable scientific standard, however I have significant reservations, as outlined above.

# Response

**Published:** 2020-08-20

**Authors:** Laura O'Connor

**Affiliations:** National University of Ireland, Galway, Ireland

Thank you for your review of our paper. We greatly appreciate the time you have taken, and feel your feedback will improve the clarity of our protocol.

We have changed the title to include that this protocol is for a systematic review.

In terms of the topic of interest/inclusion criteria, we agree that both are very specific. As this protocol is for a review commissioned by a department of the Health Service Executive, these objectives were laid out in the initial tender documents and represent specific areas of interest to the HSE for these specific guidelines. While this does narrow the scope of this review, it ensures that the results will be aligned with the need the eventual guidelines is meant to address. 

As for the search strategies, our searches were quite lengthy, given the specific interests laid out by the HSE. As such we made the decision to include a sample search (for CINAHL, which is representative of the searches for other databases) as extended data, as we found it challenging to accurately represent the breadth of the search and number of descriptors in any other way.

Thank you again for your time and feedback.
